# Genetic mutations in *pfcrt* and *pfmdr1* at the time of artemisinin combination therapy introduction in South Pacific islands of Vanuatu and Solomon Islands

**DOI:** 10.1186/1475-2875-13-406

**Published:** 2014-10-15

**Authors:** Karryn J Gresty, Karen-Ann Gray, Albino Bobogare, George Taleo, Jeffrey Hii, Lyndes Wini, Qin Cheng, Norman C Waters

**Affiliations:** Australian Army Malaria Institute, Enoggera, Brisbane, Queensland Australia; QIMR Berghofer Medical Research Institute, Brisbane, Queensland Australia; Malaria and Vector Borne Diseases Control Program, Ministry of Health, Honiara, Solomon Islands; Vector Borne Disease Control Program, Ministry of Health, Port Vila, Vanuatu; School of Public Health, Tropical Medicine and Rehabilitation Sciences, James Cook University, Townsville, Queensland Australia; Walter Reed Army Institute of Research, Malaria Vaccine Branch, Military Malaria Research Program, Silver Spring, Maryland USA

**Keywords:** *Plasmodium falciparum*, Chloroquine, *pfcrt*, Microsatellite markers, Surveillance, Molecular markers, Vanuatu, Solomon Islands

## Abstract

**Background:**

Chloroquine (CQ), alone or in combination with sulphadoxine-pyrimethamine, was widely used for the treatment of *Plasmodium falciparum* and *Plasmodium vivax* for several decades in both Vanuatu and Solomon Islands prior to the introduction of artemether-lumefantrine (AL) in 2008. However, the effect of chloroquine selection on parasite population, which may affect the efficacy of lumefantrine or other partner drugs of artemisinin, has not been well assessed. This study aims to provide baseline data on molecular markers (*pfcrt* and *pfmdr1)*, along with the origins of *pfcrt,* prior to the introduction of AL.

**Methods:**

Blood spots were obtained from epidemiological surveys conducted on Tanna Island, Tafea Province, Vanuatu and Temotu Province, Solomon Islands in 2008. Additional samples from Malaita Province, Solomon Islands were collected as part of an artemether-lumefantrine efficacy study in 2008. *Plasmodium falciparum pfcrt* and *pfmdr1* genes were examined for polymorphisms. Microsatellite markers flanking *pfcrt* were also examined to ascertain origins of CQ resistance.

**Results:**

*Pfcrt* analysis revealed 100% of parasites from Tafea Province, Vanuatu and Malaita Province, Solomon Islands and 98% of parasites from Temotu Province, Solomon Islands carried the K76**T** polymorphism that confers CQ resistance. Comparison of *pfcrt* allelic patterns and microsatellite markers flanking *pfcrt* revealed six haplotypes with more than 70% of isolates possessing haplotypes very similar to those observed in Papua New Guinea. The dominant (98.5%) *pfmdr1* allele across all island groups was **Y**Y**C**ND.

**Conclusions:**

Prior to the introduction of AL in the Solomon Islands and Vanuatu, *P. falciparum* isolates possessed point mutations known to confer CQ resistance and possibly associated with a decreased susceptibility to quinine and halofantrine, but an increased susceptibility to artemisinin and lumefantrine. Overall, *pfcrt* allelic types and the flanking microsatellite markers exhibited similarities to those of Papua New Guinea, suggesting these parasites share a common ancestry. The current use of AL for both *P. falciparum* and *P. vivax* infections will enable changes in these markers, in the absence of CQ pressure, to be monitored.

## Background

Chloroquine-resistant (CQR) *Plasmodium falciparum* parasites arose in the late 1960s and have since spread throughout most malaria-endemic regions rendering chloroquine (CQ) ineffective in many areas. The spread of CQ resistance has been attributed to the resurgence of malaria morbidity and mortality during the 1990s [1, 2]. In 2001, the World Health Organization (WHO) recommended the use of artemisinin-based combination therapy (ACT) for treatment of uncomplicated *P. falciparum* malaria [3]. Following this recommendation, the South Pacific countries of Vanuatu and Solomon Islands introduced ACT in 2008 [4].

CQ resistance in *P. falciparum* has been reported to associate with mutations in the parasite CQ resistance transporter gene *(pfcrt)* located on chromosome 7. Molecular studies demonstrated that resistance to CQ results from a series of mutations in *pfcrt,* of particular importance is the mutation causing a change from lysine (K) to threonine (T) at amino acid 76 [5, 6]. The K76**T** and 11 other amino acids have been used as molecular markers for studying the evolution of CQ resistance. Various combinations of *pfcrt* mutations have been identified in different geographical locations and have been linked to past drug exposure [7].

Worldwide, there has been at least five major independent geographical origins of CQR *pfcrt* alleles. Initially four lineages were identified; one each from Indochina/Africa (CVIET) and Melanesia (SVMNT), and two from South America (SVMNT) [8–10]; a fifth lineage was later identified from the Philippines (C/SVMNT) [11, 12]. Furthermore, SVMNT has also been linked with historical amodiaquine use [7]. The origins of various *pfcrt* alleles have been mapped using microsatellite markers flanking *pfcrt* [9, 10, 12]. Microsatellites (MS) are short, simple, sequence repeats, and are prolific in the *P. falciparum* genome. When drug pressure exists in a parasite population, mutations that are advantageous to the parasite, for example those leading to drug resistance, are selected and propagated throughout the population. The MS markers that flank these resistance genes can also be propagated throughout the population via a form of genetic ‘hitch-hiking’ [9]. This leads to a reduction in the variation of allele frequency of the MS markers closest to the locus under selection [9].

*Pfcrt* mutations can also affect parasite susceptibilities to other anti-malarial drugs. For example, wild type *pfcrt* alleles confer a reduced susceptibility to lumefantrine, while mutant alleles can result in increased sensitivity to lumefantrine [13]. Transfection experiments have demonstrated the K76**T** mutation as the main determinant of *pfcrt-*dependent lumefantrine susceptibility [13]. Similarly, susceptibility levels of amodiaquine have been found to vary depending on the geographic origin of the mutant *pfcrt* alleles [7].

The multidrug resistance gene *(pfmdr1)* coding for P-glycoprotein homologue-1 (pgh-1) located on chromosome 5 [14–16] was also implicated in CQ resistance. Mutations in *pfmdr1* result in changes at amino acids 86, 184, 1034, 1042, and 1246, and these changes have been associated with CQR in laboratory clones [14] although their associations with CQR were strain specific [15] *.* These polymorphisms in the *pfmdr1* gene were also shown to affect parasite susceptibilities to structurally diverse anti-malarial compounds, for example quinine, halofantrine, mefloquine, CQ, and artemisinin *in vitro* [15, 17] *.* Additionally, an increase in copy number of the *pfmdr1* gene was reported to play a role in reduced parasite susceptibility to artemisinin derivatives as well as mefloquine, halofantrine and quinine [18–20].

Both *P. falciparum* and *Plasmodium vivax* are endemic to the Solomon Islands and Vanuatu (except for the islands of Aneityum and Futuna which are malaria-free). CQ was used as first-line therapy, alone or with sulphadoxine-pyrimethamine (SP), for both *P. falciparum* and *P. vivax* infections until 2008. Reduced susceptibility of *P. falciparum* parasites to CQ was first described in the Solomon Islands in 1980, initially occurring in Western, Central, Guadalcanal, and Malaita Provinces, extending countrywide in later years [21, 22]. In 2001, as the 28-day CQ treatment failure rate reached 67% [22] Solomon Islands replaced CQ with CQ plus SP as first-line therapy for uncomplicated falciparum malaria. In Vanuatu, resistance to CQ was first reported in 1987, following this the Vanuatu Ministry of Health introduced a new treatment regime in 1994, changing the treatment of uncomplicated *P. falciparum* infection from CQ monotherapy to a combination therapy of CQ and SP, while CQ remained the treatment for *P. vivax* infections [22]. A therapeutic efficacy study conducted in 2001 revealed a 16% treatment failure rate for CQ plus SP [22]. Despite these clinical reports, there is limited molecular data published on the prevalence of CQR malaria in Vanuatu and the Solomon Islands and their origins.

Vanuatu and the Solomon Islands are presently intensifying malaria control countrywide and progressing towards malaria elimination in targeted provinces. The success of eradicating malaria is dependent on many factors, not least of which is overcoming the challenge of drug resistance; malaria epidemiology is an integral component of any elimination strategy. Knowledge of baseline drug-resistant genotypes and their changes over time in parasite populations is critical to monitoring emergence of drug resistance and informing treatment policy. In 2008 artemether-lumefantrine (AL) was introduced to treat uncomplicated *P. falciparum* infections in both Vanuatu and the Solomon Islands. ACT is currently the first-line treatment for vivax malaria in both countries [4]. It is important to document parasite molecular drug resistance profiles at the time of AL introduction and use this baseline information for detection of future changes under AL pressure. Furthermore, the baseline information may also inform parasite susceptibility to companion drugs of ACT.

In this study, the occurrence and prevalence of mutations in *pfcrt* and *pfmdr1* was examined. Samples used in this study were collected from villages across several isolated groups of islands from Vanuatu and the Solomon Islands in 2008. The origins of CQ resistance was also investigated by analysing the MS loci surrounding the *pfcrt* gene.

## Methods

### Study areas and sample collection

Samples used in this paper were collected in three separate studies. Two sets were obtained from epidemiological surveys conducted (2008) during the wet seasons in:

1. Tafea Province, Vanuatu. This was a school-based, mass blood survey of children (2-12 years) where 73% of all children both febrile and non-febrile were assessed for malaria infection.
2. Temotu Province, Solomon Islands. This was a village-based mass blood survey encompassing all ages where 50.2% of the provincial population both febrile and non-febrile were assessed for malaria infection.

The geographic locations and information regarding the demographics of the populations surveyed and consent process for both these surveys have been published previously [23].

The third set of samples was from an AL (Coartem™, Novartis) therapeutic efficacy study conducted (June to October 2008) in the Province of Malaita, Solomon Islands (Wini *et al.,* unpublished). Febrile patients (2-68 years) attending Auki town clinic and Kilu’Ufi General Hospital were recruited by the Ministry of Health staff, Solomon Islands in collaboration with WHO. Malaita Province lies approximately 9°S 161°E and is the second largest and most densely populated Province of the Solomon Islands. In these areas malaria transmission is perennial with peaks between November and August.

Across all study sites blood samples were collected by finger prick. A blood spot (20-30 μL) was air-dried onto filter paper (Whatman No 3), sealed in individual plastic bags containing desiccant and stored at room temperature until further processing.

### Parasite DNA extraction and PCR speciation

Extraction of genomic DNA from blood on filter paper and PCR to determine *Plasmodium* species has been described previously [23].

### Parasite speciation by multiplex PCR

*Plasmodium* speciation was performed using species-specific primers in a multiplex PCR as previously described [24] to identify *P. falciparum, P. vivax, Plasmodium ovale* or *Plasmodium malariae* infections in blood samples.

### Mutations in *pfcrt*

The extracted DNA was used as the template for PCR amplification of *pfcrt*. Initially PCR/RFLP analysis [25] was used to determine isolates exhibiting the K76**T** substitution indicative of CQ resistance. Random subsets of these samples (17 from Tafea, 50 from Temotu and 31 from Malaita Provinces) were selected for further analysis of additional codons. DNA fragments covering 12 known mutations in *pfcrt* were amplified by nested PCR as described previously [26]. For Tafea and Temotu Provinces, in addition to codon 76, 11 other codons were examined (72, 74, 75, 97, 144, 160, 220, 271, 326, 356, and 371), only four additional codons (72, 74, 75, and 97) were determined for samples from Malaita Province.

### Mutations in *Pfmdr1*

Random subsets of samples (17 from Tafea, 37 from Temotu and 31 from Malaita Provinces) were selected for sequence analysis. The extracted DNA was used as the template for PCR amplification of DNA fragments covering five amino acid codons in *pfmdr1* (86, 184, 1034, 1042, and 1246) known to affect drug resistance levels as described previously [27].

### DNA sequencing

All PCR amplifications generating a single product were incubated with ExoSAP-IT^R^ (USB, Cleveland, OH, USA) to remove excess primers and nucleotides according to the manufacturer’s protocol and then sequenced. In cases where PCR products contained multiple bands, a single band of expected size was excised and purified using a NucleoSpin^R^ extraction kit (Macherey-Nagel) and then sequenced. Sequencing was performed using a Big Dye Terminator kit (v.3.1) on an automated DNA sequencer, ABI 3100 system, at the QIMR Berghofer Medical Research Institute Scientific Services Analytical Facility.

### *Plasmodium falciparum*microsatellite genotyping

For selected samples five MS markers flanking the *pfcrt* gene, B5M77 (-20 kb), 2E10 (-5 kb), PE12A (+6 kb), 2H4 (+22 kb), and PE14F (+106 kb), were amplified and analysed as described previously [9]. An additional six *P. falciparum* laboratory isolates (AN001, AN018, Dd2, C2B, PH1, and 7G8) obtained from diverse geographical regions were analysed as reference strains [12]. MS sizes in samples were compared to these reference strains and calibrated accordingly.

## Results

### Prevalence of mutations in *pfcrt*

Sequences of *pfcrt* were examined and the prevalence of mutations determined for the three island groups. Amino acid substitutions were identified at codons 72, 76, 220, 326, and 356 whereas wild type amino acids were observed at codons 97, 144 and 160. Initial PCR/RFLP analysis revealed 100% of parasites from Tafea Province, Vanuatu and Malaita Province, Solomon Islands and 98% of parasites from Temotu Province, Solomon Islands carried the K76**T** substitution indicative of CQR (Table 1). Only one sample from the village of Neo on Santa Cruz Island, Temotu Province exhibited the wild type lysine (K) at codon 76 as well as cysteine (C) at codon 72, however this sample had the A220**S** substitution (Table 2). All three island groups possessed *pfcrt* C72**S** and K76**T** substitutions at 94 and 98% or greater, respectively. Likewise, polymorphisms A220**S**, N326**D**/**S** and 356 **L**/**T** were 91% or greater for Tafea and Temotu Provinces (these codons were not examined for Malaita Province). All other codons examined indicated predominance for wild type amino acids M74, N75, H97, A144, L160, Q271, and R371 (Table 1).

**Table 1:** **Prevalence (%) of amino acid substitutions in PfCRT in** ***Plasmodium falciparum*** **samples collected from Tafea Province, Vanuatu and Temotu and Malaita Provinces, Solomon Islands**

Country	Province	n	% substitutions at amino acid positions in PfCRT											
			72	74	75	76	97	144	160	220	271	326	356	371
Vanuatu	Tafea	17	94	6	6	100	0	0	0	100	43	100	94	47
Solomon Islands	Temotu	50	98	0	0	98	0	0	0	98	0	91	98	22
	Malaita	31	100	0	0	100	0	*	*	*	*	*	*	*

PfCRT = *Plasmodium falciparum* chloroquine resistance transporter.

* = not determined.

**Table 2:** ***Pfmdr1*** **and** ***pfcrt*** **genotypes and MS markers flanking** ***pfcrt*** **in** ***Plasmodium falciparum*** **samples collected from Tafea Province, Vanuatu and Temotu and Malaita Provinces, Solomon Islands compared to reference samples**

Origin (no. isolates)	n	H	%	MS^a^marker size (bp)		AA positions in *Pfcrt* ^b^	MS^c^marker size (bp)			AA positions in *Pfmdr1* ^*d*^
				B5M77	2E10		PE12A	2H4	PE14F	
Vanuatu Tafea (n = 17)	5	1	29.4	147	174	**S**MN**T**HAL**S**Q**DL**R	328	184	142	**Y**Y**C**ND
	1	1	5.9	147	174	**S**MN**T**HAL**S**Q**DLI**	314	184	142	**Y**Y**C**ND
	1		5.9	*	*	**S**MN**T**HALS***R	*	*	*	**Y**Y**C**ND
	2	2	11.7	157	156	**S**MN**T**HALSE*TI	*	201	148	YF*ND
	5		29.4	*	*	**S**MN**T**HALS**TI	*	*	*	**Y**Y**C**ND
	1		5.9	*	*	**S**MN**T**HALS**TR	*	*	*	**Y**Y**C**ND
	1		5.9	*	*	**S**MN**T**HALS*STR	*	*	*	**Y**Y**C**ND
	1		5.9	*	*	C**IET**HALSE*TR	*	*	*	**Y**Y**C**ND
Solomon Islands Temotu (n = 50)	23	3	64.7	149	174	**S**MN**T**HAL**S**Q**DL**R	328	192	139	**Y**Y**C**ND
	4	4	8.9	149	174	**S**MN**T**HAL**S**Q**DL**R	328	184	134	**Y**Y**C**ND
	1	4	2.9	149	174	**S**MN**T**HAL**S**Q**DL**R	328	184	136	**Y**Y**C**ND
	1	4	2.9	149	174	**S**MN**T**HAL**S**Q**DL**R	328	184	142	**Y**Y**C**ND
	1		2.9	*	*	**S**MN**T**HAL**S**Q**DL**R	*	*	*	**Y**Y**C**ND
	1	4	33.3	149	174	**S**MN**T**HAL**S**Q**DT**R	328	184	134	**Y**Y**C**ND
	1		2.9	*	*	**S**MN**T**HAL**S**Q**DT**R	*	*	*	**CND
	7		5.9	*	*	SMN**T**HAL**S**Q**STI**	*	*	*	**CND
	2		5.9	*	*	SMN**T**HAL**S**Q*TI	*	*	*	**CND
	1	5	2.9	140	166	CMNKHAL**S**QNIR	314	184	148	**CND
	2	3	28.5	149	174	**S**MN**T**HAL**S**Q**DL**R	328	192	139	**Y**Y**C**ND
	1	3	14.2	149	174	**S**MN**T**HAL**S**Q**DLI**	328	192	139	**Y**Y**C**ND
	1		14.2	*	*	**S**MN**T**HAL**S**Q**DL**R	*	*	*	**Y**Y**C**ND
	1		16.7	*	*	**S**MN**T**HAL**S**Q**SL**R	*	*	*	**CND
	1		14.2	*	*	**S**MN**T**HAL**STR	*	*	*	**Y**Y**C**ND
	1		14.2	*	*	**S**MN**T**HAL**STI	*	*	*	**Y**Y**C**ND
	1		14.2	*	*	**S**MN**T**H**A**TR	*	*	*	**CND
Solomon Islands Malaita (n = 31)	9	3	29	149	174	**S**MN**T**HAL**S**Q**DL**R	328	192	139	**Y**Y**C**ND
	11	3	35.5	149	174	**S**MN**T**HAL**S**Q**DLI**	260	192	139	**Y**Y**C**ND
	4	4	12.9	149	174	**S**MN**T**HALS***R	328	184	145	**Y**Y**C**ND
	1	6	3.2	149	154	**S**MN**T**HALSE*TI	328	192	142	**Y**Y**C**ND
	3	1	9.7	147	174	**S**MN**T**HALS**TI	328	184	142	**Y**Y**C**ND
	3		9.7	*	*	**S**MN**T**HALS**TR	*	*	*	**Y**Y**C**ND
World reference Samples	Brazil 7G8			151	190	**S**MN**T**HAL**S**Q**DL**R	314	194	142	
	Philippines PH1			149	182	CMN**T**H**TY**AQ**DL**R	314	228	136	
	Thailand Dd2			149	170	C**IET**HAL**SESTI**	314	204	145	
	Thailand C2B			149	170	C**IET**HAL**SESTI**	314	184	148	
	Solomon Island N18			149	174	**S**MN**T**HAL**S**Q**DL**R	328	184	142	
	PNG AN001			149	174	**S**MN**T**HAL**S**Q**DL**R	328	184	142	
	PNG AN018			149	174	**S**MN**T**HAL**S**Q**DL**R	328	192	139	

Note: Boldface amino acids represent the mutated state of that codon.

^a^MS microsatellite upstream of *pfcrt*:B5M77 (-20 kb) and 2E10 (-5 kb).

^b^Amino acid (AA) positions in *pfcrt* are 72, 74, 75, 76, 97, 144, 160, 220, 271, 326, 356, 371.

^c^MS microsatellite downstream of *pfcrt*: PE12A (+6 kb), 2H4 (+22 kb) and PE14F (+106 kb).

^d^Amino acid positions in *pfmdr1* are 86, 184, 1034, 1042, 1246.

*not determined.

** or *** indicates 2 or 3 AA positions were not determined

### *Pfcrt*allelic types

Sequence analysis of *pfcrt* at codons 72, 74, 75, 76, and 97 were completed for all samples investigated (17 from Tafea, 50 from Temotu and 31 from Malaita Provinces) and results revealed that 96/98 (98%) to be allelic type **S**MN**T**H. One other mutant allelic type C**IET**H was identified in a sample from Vanuatu while a wild type allele CMNKH was identified in one sample from Temotu Province, Solomon Islands.

In conjunction with these five codons, additional PfCRT amino acids 144, 160, 220, 271, 326, 356, and 371 were examined in samples from Tafea Province, Vanuatu and Temotu Province, Solomon Islands. Amino acids were determined at all 12 codons investigated for 48 isolates from Tafea Province, Vanuatu and Temotu Province, Solomon Islands, with remaining isolates having incomplete amino acid identification at one or more codons. Of the 51 completed sequences a predominant allele, **S**MN**T**HAL**S**Q**DL**R**,** was identified in 38 isolates (74.5%), with the remainder being predominantly polymorphic at codons 271, 326 and 356. This predominant allele was found in 83% (5/6) and 73% (33/45) of samples from Tafea Province, Vanuatu and Temotu Province, Solomon Islands, respectively. One sample from Port Resolution on Tanna Island, Tafea Province possessed allele C**IET**HALSE*TR (unidentified amino acids are represented by asterisk) similar to the dominant allele in samples originating in Thailand. In Tafea Province, Vanuatu, 33% (3/9) of samples possessed the Q271**E** mutation most commonly seen in Thailand. Interestingly, this mutation was not detected in Temotu Province, Solomon Islands despite twice the number of samples being examined. Furthermore, one CQR isolate (**S**MN**T**H**A**TR) from the village of Ngamubulo, situated on the Reef Islands, Temotu Province, Solomon Islands did not exhibit the A220**S** polymorphism generally associated with CQR alleles. Observed allelic types and their prevalence in the different island groups are summarized in Table 2.

### Microsatellite haplotypes

Throughout the island provinces, genotyping of five MS markers flanking *pfcrt* revealed six haplotypes (based on similarity of 4/5 markers), H1-H6. The prevalence of each haplotype is different between the island groups (Table 2 and Table 3).

**Table 3:** **Prevalence of MS haplotypes between different island groups**

Country	Province	Prevalence (%)					
		H1	H2	H3	H4	H5	H6
Solomon Islands	Temotu	0.0	0.0	76.5	20.6	2.9	0.0
	Malaita	7.1	0.0	71.4	14.3	0.0	7.1
Vanuatu	Tafea	75.0	25.0	0.0	0.0	0.0	0.0

In Temotu Province, Solomon Islands, 34 of the 50 samples were successfully typed at all five loci and resulted in three haplotypes, of these 76.5% (26/34) and 20.6% (7/34) were H3 and H4, respectively, representing 97.1% of the parasite population (Table 3). These haplotypes have been reported in PNG (AN018 and AN001 groups). Interestingly, one sample from the village of Neo on Santa Cruz Island possessing a single A220**S** polymorphism, exhibited the Southeast Asian *pfcrt* allelic type and also had a MS pattern matching that of the Thailand C2B group at all three downstream MS loci.

In Malaita Province, Solomon Islands, 28 of the 31 isolates were successfully typed at all five loci and revealed four MS haplotypes (based on similarity of 4/5 MS markers). Of these 71.4 (20/28) and 14.3% (4/28) were H3 and H4, respectively, representing 85.7% of parasite population (Table 3). Three samples were classified as H1, possessing a variation at an upstream locus compared to H3 and H4. One sample was determined H6, also having a variation at an upstream locus.

In Tanna Island, Vanuatu, eight of 17 isolates were successfully typed at all five loci resulting in two patterns (based on similarity of 4/5 MS markers). Of these 75% (6/8) were determined as H1 (Table 3) showing close identity to those observed for the Solomon N70 group [12]. The two remaining samples, from the village of Fetukai, had unique MS sizes at three loci, while locus PE14F was observed to be identical to samples from the Thailand C2B group. These two samples were classified as H2. Interestingly, despite having the **S**MN**T**HAL in PfCRT, they also possessed a change in amino acid from glutamine (Q) to glutamic acid (E) at codon 271, a characteristic common to both groups of samples originating from Thailand (Dd2 and C2B).

### Prevalence of mutations in *pfmdr1*

Sequences of *pfmdr1* were analysed and the prevalence of mutations in the three island groups determined (Table 4). Amino acid substitutions were identified at codons 86, 184 and 1034 while only wild type codons were observed at 1042 and 1246. Polymorphisms N86**Y** and S1034**C** were present in 100% of samples from both countries, while one sample from Vanuatu possessed an additional mutation Y184**F**. The dominant *pfmdr1* allele across all island groups was **Y**Y**C**ND. The observed allelic types in the different island groups are summarized in Table 2.

**Table 4:** **Prevalence (%) of amino acid substitutions in PfMDR1 in** ***Plasmodium falciparum*** **samples collected from Tafea Province, Vanuatu and Temotu and Malaita Provinces, Solomon Islands**

Country	Province	n	% substitutions at amino acid positions in PfMDR1				
			86	184	1034	1042	1246
Vanuatu	Tafea	17	100	6	100	0	0
Solomon Islands	Temotu	37	100	0	100	0	0
	Malaita	31	100	0	100	0	0

PfMDR1 = *Plasmodium falciparum* multidrug resistance protein 1.

## Discussion

CQ has been used widely in the South Pacific for many years and has applied a strong selection pressure on parasite populations, which may affect the efficacy of ACT. Vanuatu and the Solomon Islands are among the few countries where ACT is currently used for treatment of both *P. falciparum* and *P. vivax* malaria. Understanding the parasite drug resistance profile at the time of ACT introduction allows for detection of changes in future parasite populations. It will also enable a better understanding of potential changes in drug resistance profiles for both species in the absence of CQ drug pressure. In this paper, the baseline data on *pfcrt* and *pfmdr1* was investigated at the time of AL introduction.

Overall results of this study indicated a dominance of the **S**MN**T** allele across all island groups. Amino acid analysis indicated 100% of samples examined from Tafea Province, Vanuatu and Malaita Province, Solomon Islands, and 98.8% of samples from Temotu Province, Solomon Islands possessed the K76**T** polymorphism indicative of CQ resistance, and possibly impaired quinine and halofantrine susceptibility. This also demonstrates that the CQR *pfcrt* allele is at fixation in these three island group populations of Vanuatu and Solomon Islands. The high prevalence of CQR parasites in these two countries is not surprising given CQ monotherapy was the primary chemotherapeutic drug used to treat uncomplicated *P. falciparum* malaria for several decades, and until relatively recently, was also the treatment of choice for *P. vivax* infections. Although CQ has not been used in Vanuatu and the Solomon Islands as a monotherapy since 1994 and 1991, respectively, it was still used in combination therapy for treatment of *P. vivax* infections. This continued CQ pressure provides a reasonable explanation for the prevalence of the 76 **T** mutation in these parasite populations. Interestingly, published *in vivo* and *in vitro* studies suggest the effectiveness of lumefantrine is enhanced in *P. falciparum* parasites carrying the *pfcrt* K76**T** mutation [13]. This suggests that lumefantrine is an ideal ACT partner drug to use in Vanuatu and the Solomon Islands as both these regions exhibit high levels of CQ resistance.

All samples exhibiting the K76**T** polymorphism also possessed additional mutations occurring in at least one of the following codons 220, 326, 356, and 371. While **S**MN**T**HAL**SQDL**R was predominant on all three island groups, variation in *pfcrt* genotypes exists not only between these two countries but also within island groups. In Temotu Province, one of 50 samples analysed retained a wild type K76 in conjunction with a single A220**S** polymorphism (genotype CMNKHAL**S**QNIR). Although the A220**S** polymorphism in *pfcrt* has been shown to increase the level of CQ resistance when co-existing with K76**T**, it is unlikely that a single A220**S** polymorphism is sufficient to confer CQR [28]. In contrast, another isolate from the village of Ngamubulo situated on the Reef Islands did not exhibit the A220**S** substitution generally associated with CQR alleles having a genotype of **S**MN**T**H**A**TR. The occurrence of the A220 in combination with the K76**T** mutation, although not common, has been previously documented in isolates from the Philippines, as well as India and China [12, 29, 30]. Q is found at codon 271 in all samples, including reference samples, except those originating in Thailand, which predominantly contain an E at this position. Interestingly, 43% of samples from Tafea Province, Vanuatu contained the Q271**E** substitution. At this point the drug pressure that selects for this polymorphism is not understood; however, it may be indicative of changes in drug sensitivity profiles and/or the subsequent sweep of drug resistant alleles throughout the region, hence this codon may be a key amino acid for future surveillance studies.

Overall, results indicated that 74.5% (38/51) of samples collected from Tafea Province, Vanuatu and Temotu Province, Solomon Islands, for which all 12 *pfcrt* codons could be determined, had a *pfcrt* allelic type identical to those from Papua New Guinea (PNG). This may indicate a gene flow of parasite alleles between these island groups and suggests that malaria drug resistance in these provinces is similar to PNG. At this time it is unclear if clinical drug resistance differences exist between Tafea Province, Vanuatu and Temotu Province, Solomon Islands, however *pfcrt* analysis of *P. falciparum* samples collected from these locations predict similar drug susceptibilities. Furthermore, the **S**MN**T**H allelic type of *pfcrt* in these island groups suggests a past parasite exposure to amodiaquine (AQ) [7] although no evidence of AQ use in either Vanuatu or the Solomon Islands is evident. AQ was however introduced in PNG in 1966 and given its close geographical proximity, the likelihood of drug resistance genotypes becoming established in the neighbouring countries of Solomon Islands and Vanuatu seems predictable.

Analysis of five MS markers flanking *pfcrt* was undertaken to determine whether parasites harbouring mutations that conferred CQR evolved locally or originated via gene flow from other countries. Overall, 70 of 98 samples from three island groups were typed revealing six MS haplotypes. There were difficulties in obtaining MS data for many of the samples analysed, particularly from Tanna Island and Temotu Province; this was possibly due to low levels of parasitaemia in these samples as these were obtained from cross-sectional surveys of villagers most of whom were asymptomatic and had low parasitaemia at time of survey. Interestingly, also in Tafea and Temotu Provinces there was a high incidence of asymptomatic infections. In Tafea Province only 28% of children that were slide positive for malaria were febrile while in Temotu Province only 14% of all ages tested that were slide positive for malaria were febrile [31]. This suggests in these regions that a high proportion of people infected with malaria were asymptomatic. In comparison 63% of samples collected from Malaita Province were from febrile patients attending the clinic or hospital. This implies that only sampling from symptomatic individuals (ie. those attending clinics or hospitals) has the potential to miss many positive malaria cases and this may lead to undetected genotypes circulating within populations.

Interestingly, the distribution and prevalence of the six haplotypes were different between the island groups. H3 and H4 were predominant haplotypes in both Temotu and Malaita Provinces, Solomon Islands. Both these haplotypes were also dominant types in PNG [10, 12]. The MS and the *pfcrt* typing results strongly indicate that CQR parasites in two different provinces of Solomon Islands share the same origin as those from PNG and may have resulted from a spread via PNG. Minor haplotypes were also observed in these two island groups, H5 was present in Temotu while H1 and H6 were in Malaita Province. H5 has a distinct MS pattern upstream to *pfcrt* and has a wild type *pfcrt* representing a minor CQ-sensitive parasite population*.* The minor types H1 and H6, have small variations to H3 and H4, and may have resulted from genetic drift.

In Tafea Province, Vanuatu, H1 appears to be the dominant haplotype albeit the sample number is relatively small. Although there is variation, it is closely related to H4, which has been reported in samples from Guadalcanal, Solomon Islands previously [12]. Therefore, parasites in this province may have spread from Solomon Islands or PNG. The haplotype H2 (n = 2) observed in this location may have been generated from a recombination between Southeast Asian and PNG parasites. The Q271**E** polymorphism detected in these two parasites, which is common to Southeast Asian CQR parasites, and the presence of Southeast Asian CQR *pfcrt* allele in the province support this hypothesis.

Interactions between *pfcrt* and *pfmdr1* mutations and their combined effect on drug resistance are not clear. Like *pfcrt*, direct evidence has demonstrated that point mutations in *pfmdr1* can confer resistance to CQ, mefloquine, quinine, and halofantrine [15]. It has been suggested that the response of CQR falciparum parasites to CQ is associated not only with the *pfcrt* genotype but also the *pfmdr1* mutations with which they are coupled [7]. In this study the dominant (98.5%) *pfmdr1* allele across all island groups was **Y**Y**C**ND, only one isolate from Vanuatu possessed a YF*ND allele. Studies have suggested that the Y184**F** mutation is associated with low-level resistance to AL [32] but as yet there has been no consensus for the role it plays in CQ resistance. The change in amino acid from asparagine to tyrosine N86**Y** has been associated with CQ resistance in some investigations [33–36] but not others [37–40]; this polymorphism has also been implicated in playing a modulating role in reducing the susceptibility of parasites to amodiaquine [41–44], a 4-aminoquinoline drug structurally related to CQ. A clinical trial in Zanzibar, in which *pfcrt* K76**T** and *pfmdr1* N86**Y** frequencies were determined before AL administration and then again in all recurrent parasites during a follow-up period of 42 days, showed a significant increase in the occurrence of *pfmdr1* N86, suggesting N86 as a potential marker of lumefantrine resistance *in vivo* [45]. Thus, future surveillance studies in Vanuatu and the Solomon Islands, post implementation of AL, will be of interest to see if there are similar changes in the frequency of *pfmdr1* mutations.

## Conclusions

This study provides a clear indication of widespread CQ resistance in both the Solomon Islands and Vanuatu at the time of AL introduction. Overall the *pfcrt* genotypes and the MS markers flanking them in samples from Tafea Province, Vanuatu, and Temotu and Malaita Provinces, Solomon Islands exhibited similarities to those of PNG suggesting that malaria drug resistance profiles are also similar between these countries. The recent introduction and use of AL throughout this region as first-line treatment for both *P. falciparum* and *P. vivax* infections, warrants continued surveillance not only to monitor baseline but also to observe correlative changes in the *pfcrt* allele in the absence of CQ pressure.
